# A community-based education programme to reduce insecticide exposure from indoor residual spraying in Limpopo, South Africa

**DOI:** 10.1186/s12936-019-2828-5

**Published:** 2019-06-14

**Authors:** Brenda Eskenazi, David I. Levine, Stephen Rauch, Muvhulawa Obida, Madelein Crause, Riana Bornman, Jonathan Chevrier

**Affiliations:** 1 Center for Environmental Research and Children’s Health (CERCH), School of Public Health, University of California at Berkeley, 1995 University Ave. Suite 265, Berkeley, CA 94704 USA; 2 Haas School of Business, University of California at Berkeley, Berkeley, CA USA; 3 University of Pretoria Institute for Sustainable Malaria Control, School of Health Systems and Public Health, University of Pretoria, Pretoria, South Africa; 4 Department of Urology, University of Pretoria, Pretoria, South Africa; 5 Department of Epidemiology, Biostatistics and Occupational Health, McGill University, Montréal, QC Canada

**Keywords:** Community education, Dramatic presentation, Indoor residual spraying, Insecticide exposure, Malaria prevention, Questionnaire, Vhembe, South Africa

## Abstract

**Background:**

Indoor residual spraying (IRS), the coating of interior walls of houses with insecticides, is common in malaria-endemic areas. While important in malaria control, IRS potentially exposes residents to harmful insecticides. The World Health Organization recommends steps to minimize exposure; however, no programme has focused on educating populations.

**Methods:**

A dramatic presentation and song were developed by study personnel and performed by lay performers in order to spread awareness of the importance of IRS and to minimize insecticide exposure. Performances were staged at 16 sprayed villages in the Vhembe District of Limpopo, South Africa, at which 592 attendees completed short questionnaires before and after the performance about behaviors that might limit insecticide exposure. Overall indices of the attendees’ change in knowledge of precautions to take prior to and after spraying to prevent insecticide exposure were analyzed using hierarchical mixed models to assess the effect of the performance on change in participants’ knowledge.

**Results:**

Approximately half of attendees lived in homes that had been sprayed for malaria and 62% were female. Over 90% thought it better to allow IRS prior to the presentation, but knowledge of proper precautions to prevent exposure was low. The proportion answering correctly about proper distance from home during spraying increased from 49.4% pre-performance to 62.0% post-performance (RR = 1.26, 95% CI = 1.13, 1.41), and the proportion reporting correctly about home re-entry interval after spraying increased from 58.5 to 91.1% (RR = 1.54, 95% CI 1.35, 1.77). Attendees improved in their knowledge about precautions to take prior to and after spraying from mean of 57.9% correct to a mean of 69.7% (β = 12.1%, 95% CI 10.9, 13.4). Specifically, increased knowledge in closing cupboards, removing food and bedding from the home, covering immoveable items with plastic, and leading animals away from the home prior to spraying were observed, as was increased knowledge in sweeping the floors, proper disposal of dead insects, and discarding dirty washrags after spraying.

**Conclusions:**

A dramatic presentation and song were able to increase the attendees’ knowledge of precautions to take prior to and after spraying in order to limit their insecticide exposure resulting from IRS. This approach to community education is promising and deserves additional study.

## Background

In 2017, there were 219 million cases of malaria, resulting in about 435,000 deaths, with most deaths occurring to children under age 5 and living in sub-Saharan Africa [1]. Increased prevention and control measures have led to an overall reduction in global malaria mortality rate of about 60% since 2000 [1]. A total of 21 countries worldwide, including South Africa, were identified as likely to reach zero indigenous cases by 2020 [1]. However, South Africa is not on target to meet these World Health Organization (WHO) goals, having experienced a > 20% increase in cases between 2016 and 2017 [1]. Inadequate mosquito vector control, improved reporting, and climatic changes likely play a role in this increase [1]. In South Africa, there are three malaria-endemic provinces: Mpumalanga, KwaZulu-Natal, and Limpopo [2]. Between 2000 and 2010, Limpopo had the lowest decrease in malaria cases in South Africa [2], and between 2013 and 2014 malaria cases nearly doubled [3].

South Africa is part of the Elimination 8 (E8) Regional Initiative to jointly plan and execute a regional malaria elimination strategy for eight Southern African countries by 2030 [4]. The major components of the South Africa national policy for malaria elimination includes malaria vector control by indoor residual spraying (IRS), early detection by rapid diagnostic test (RDT), and artemisinin-based combination therapy. IRS is the systematic insecticide application to the interior walls of homes to kill malaria-infected mosquitoes as they rest. IRS with the organochlorine insecticide dichlorodiphenyltrichloroethane (DDT) has been ongoing since 1946 in malaria-endemic regions of South Africa [5]. DDT and its main breakdown product, dichlorodiphenyldichloroethylene (DDE), have long half-lives in the human body (6 years and up to 10 years, respectively) and in the environment. Thus, the Stockholm Convention banned DDT for all uses except for public health purposes due to its toxicity [6–8]. In recent years, pyrethroid insecticides have been primarily used for IRS in South Africa [9]. In Vhembe, cypermethrin was used until 2015 and deltamethrin has been used since then. It is not South African policy to rotate pyrethroids or to distribute pesticide-impregnated bed nets.

Both DDT and pyrethroids are neurotoxic in developing animals through a number of mechanisms [9–11] and both DDT [12] and some pyrethroids [13] are endocrine disruptors. Thus, although effective prevention of malaria is of critical importance to human health, there is growing concern about the potential health effects of IRS insecticides [14, 15]. Previously, we have identified in a Vhembe birth cohort associations between DDT in maternal serum collected at delivery and increased maternal hypertension [16], childhood infections [17], and larger fetal and childhood growth in girls [18, 19]. Associations have also been found between pyrethroid metabolites in maternal urine and negative sequelae in their children, including impaired neurobehavioral development [20], and decreased childhood growth in boys [19].

A key goal of South Africa’s Malaria Elimination Strategy is to ensure that by 2018, 100% of the population has adequate malaria knowledge and practice [21]. To reach this goal, One Sun Health, a non-profit organization, developed a Malaria Awareness Programme (MAP) in the northern Vhembe region of Limpopo [22]. The curriculum for MAP included innovative methods to train home-based care, over multiple sessions, on malaria transmission. Teaching methods included visual diagrams, a song to teach symptoms, a drama to teach prevention, and a graphic to teach treatment. A number of other studies around the world have also investigated the effectiveness of education programmes in increasing knowledge of malaria symptomatology, prevention, and treatment [22–26] and several have used dramatic presentations or song to deliver the educational messages [27–29]. Although best practices to reduce exposure to insecticides were outlined by the WHO [30] and the President’s Malaria initiative [31], no prior study has included in their education programme the prevention of insecticide exposure to residents from IRS.

This study examines the effectiveness of a dramatic presentation and song to convey to residents living in the Vhembe district municipality of Limpopo South Africa the importance of IRS for malaria control and ways to prevent insecticide exposure. Herein, the effectiveness of a single dramatic educational presentation is assessed in increasing knowledge of prevention of undue insecticide exposure to residents.

## Methods

### Participants

**Fig. 1:**
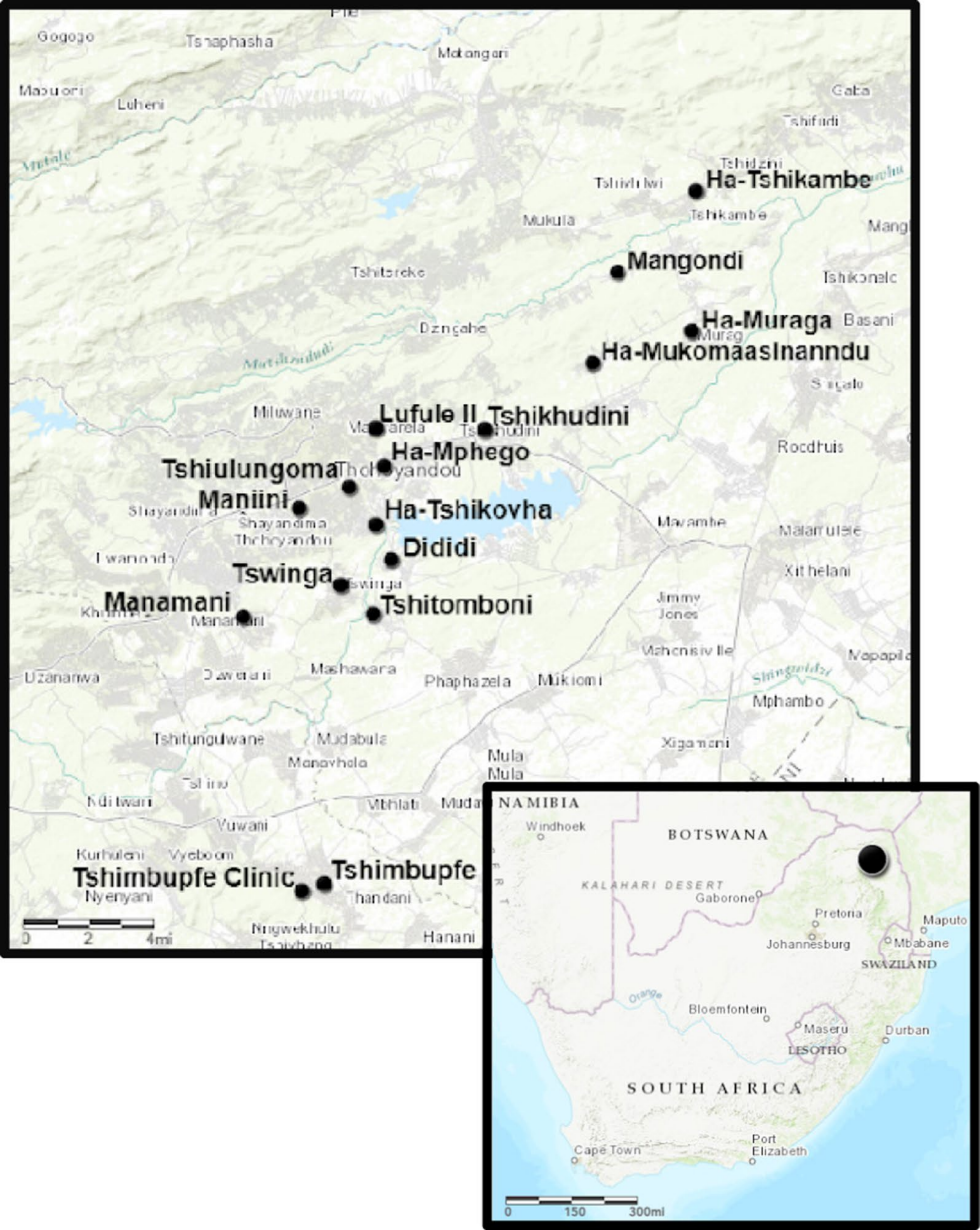
Location and names of the villages in Limpopo South Africa where skit and song were performed

Participants were adults residing in 16 villages in the Thulamela region of the Vhembe District Municipality of Limpopo Province in South Africa where IRS was conducted (Fig. 1). Participants were mainly VhaVenda and spoke TshiVenda. Research staff personally invited women (with phone calls and in person) who had participated in the Venda Health Examination of Mothers, Babies and their Environment (VHEMBE) birth cohort study [20]. In addition, they were encouraged to invite friends, family, and other residents of their villages. The meetings were also promoted with posters and word-of-mouth campaigns by VHEMBE staff and local chiefs. Attendees who were able to read and write and 18 years or older were asked to complete a questionnaire both before and immediately after the presentation. Of the 1068 adults who attended, 592 (55.4%) completed the questionnaire both pre- and post-presentation.

### Educational programme

The educational programme was conceptualized within the framework of the Health Belief Model [32, 33], a widely used value expectancy theory that aims to both predict and explain behavior. This theory suggests that people’s propensity to individual health behaviour change depends on their perceptions of: (1) their susceptibility to a health risk or condition; (2) the severity of the risk or illness; (3) the benefits of taking action; and (4) the barriers or costs of acting [33]. Some theorists also emphasize the value of a “cue to action” which may motivate individuals to take a desired step. In this study, the educational intervention (performance and/or song) is both the means of providing knowledge and a “cue to action” encouraging or further reinforcing the desired health behaviour changes, i.e., allowing indoor home spraying but practicing protective measures in advance.

**Fig. 2:**
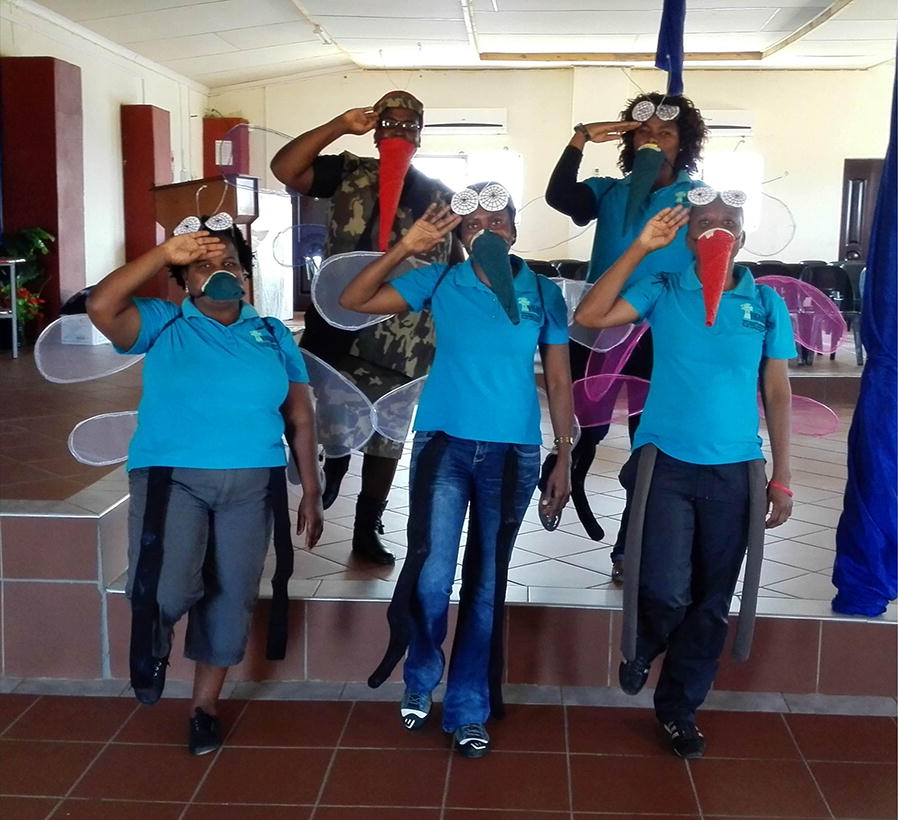
Photograph of skit performers in costume

The education programme consisted of a skit followed by a song in TshiVenda. The purpose of the skit and song was to educate the audience on the importance of IRS for malarial control and on the necessary precautions in preventing undue insecticide exposure. The skit was developed at University of California Berkeley (D.I.L.). Then, feedback was elicited from the field office staff of approximately 10 individuals. The revised skit was then presented to patients and staff at the local hospital, as well as to drama students at the University of Pretoria. The skit was then revised based on this feedback. The song was developed by the field office staff based on the information in the skit. The skit and song were performed by VHEMBE study staff in costume as mosquitos (Fig. 2) and identified several precautions to be taken before and after spraying based on best practices outlined by the 2015 WHO Report [30].

The skit told a story about a mosquito army that sets out to spread malaria to the local village (see Appendix 1 for script in English and TshiVenda). The mosquitoes grow concerned when a loving mother tells her daughter how important it is to spray their home with insecticide. The daughter then provides several arguments against spraying: It will hurt her doll and her dog. The mosquito army cheers after each objection. Then the performers ask the audience for suggestions on how to keep toys safe from the spray. The mosquito army is sad when the mother points out—and acts out—that toys should be outside the home during the spraying. Performers identify and act out the WHO-recommended pre-spraying precautions and post-spraying behaviours that reduce exposure. At the end of the presentation, they sing a song reinforcing the overall message that IRS is beneficial and that everyone should minimize insecticide exposure to themselves and their families (see Appendix 2) (see videos of skit and song: https://www.youtube.com/watch?v=lvZ1W5ZJM8Y; https://www.youtube.com/watch?v=S_gbrBxDGys). The skit and song were performed in churches or community halls; however, the video clips above were taken from performances in local schools (at which questionnaires were not collected) due to better sound quality.

### Evaluation of knowledge of precautions

Each eligible participant was asked to complete a pre- and a post-performance questionnaire. The two sets of questions were identical (see Appendix 3) except that demographic information, such as sex, age group, and whether an attendee’s home had been sprayed for malaria (regardless of whether they lived there at the time) were included in the pre-performance questionnaire. The pre- and post-performance questionnaires included questions about whether it was better to allow spraying for malaria control in their homes or not, the proper distance to stay from the home while spraying is occurring, and the length of time they should wait after spraying before reentering their homes. They were also given lists of behaviours (some helpful, some harmful, some neutral) for before and after spraying (12 each) and asked to indicate which they should do. All behaviours referred to in the questionnaire were touched upon in the skit and in the song.

Each participant was handed the pre-performance and post-performance questionnaires, with the latter folded and stapled closed so that the participant could not read it during the performance. Before the drama and song were begun the participants were asked to complete the pre-performance questionnaire. The staff then collected each of these pre-performance questionnaires. The drama and song were performed, and afterwards participants were asked to open the closed post-performance questionnaire and complete it; forms were again collected from each individual. During the completion of the questionnaires, study staff walked around to make sure that the participants would not instruct each other, copy from another, or complete the form for someone else. Instead, they were encouraged to ask staff if they needed clarification on how to complete the questionnaires; staff were not allowed to explain the meaning of the questions. Participants were asked to answer according to how they understood the questions.

No identifying information was collected. Verbal informed consent was obtained at the group level. As part of the opening remarks, the event facilitator explained the purpose of the questionnaire and emphasized that participation was voluntary. Verbal consent was implied by completion of the questionnaire. The questionnaire and consent procedures were approved by the Institutional Review Boards at the University of California, Berkeley and the University of Pretoria in South Africa.

### Statistical analysis

For questions regarding gender, age, and whether the participant’s home had been sprayed for malaria, invalid responses were set to missing. In all, there were 26 missing values for gender, 27 for age, and 38 for spray status.

Multiple-choice questions with a single correct answer were assessed using the proportion of correct responses before and after the presentation. As an overall measure, each attendee’s total percent correct was calculated for questions involving all individual pre-spraying behaviours (out of 9), all post-spraying behaviours (out of 12), and overall (out of 21). The neutral behaviours (3 prespraying behaviours having no effect on exposure, e.g., serving tea to the spray workers) were not included in the percent correct. Each specific behaviour was also assessed comparing the correctness of an attendee’s response pre- versus post-presentation.

The effect of the presentation was assessed by comparing responses before and after the presentation using hierarchical mixed models with random intercepts for the village and attendee, adjusted for age and gender. For multiple-choice questions with a single correct answer, relative risks (RR) of the probability of responding correctly were estimated, using binary models with a log link and Poisson distribution function, estimating standard errors using the Huber-White sandwich estimator [34, 35]. (Roughly, the RR represents the change in probability of a correct response, and an RR above 1 indicates an increased probability). For each attendee’s percentage of correct responses (for pre-spraying behaviours, post-spraying behaviours, and overall), mixed linear regressions were used to estimate the adjusted mean change (β) as a result of the presentation. Relative risks of the probability of responding correctly to individual questions were also assessed, using the same binary-outcome models as described above. The effects of the presentation were assessed for the entire study population, as well as for the subset of participants who reported that their homes had been sprayed for malaria control.

## Results

The number of participants returning surveys from each of the 16 villages ranged from 8 to 97 people per village (median is 24) (see Fig. 1 for a map of the villages). Nearly two-thirds of participants (61.7%) were female. Participants were fairly evenly distributed among age categories, with the largest number over 40 years. Slightly more than half of participants (54.0%) reported currently living in homes that had ever been sprayed for malaria control (whether or not they lived there at the time) (Table 1).

**Table 1:** Demographic characteristics of presentation attendees, Limpopo, South Africa, 2015–2016

	N (%)
Sex^a^	
Male	217 (38.3)
Female	349 (61.7)
Age^b^	
18–24	127 (22.5)
25–30	128 (22.7)
31–40	120 (21.2)
41 and older	190 (33.6)
Home ever sprayed for malaria?^c^	
Yes	299 (54.0)
No	209 (37.7)
Don’t know	46 (8.3)

^a^Missing 26 values

^b^Missing 27 values

^c^Missing 38 values

Table 2 presents a comparison between the pre- and post-performance responses to questions about whether to allow IRS, their location during spraying, and the timing of re-entry into the home. Almost all thought it was better to have their home sprayed pre-performance, yet this percent increased slightly post-performance, from 95.1 to 98.1% (RR = 1.03, 95% CI 1.00, 1.06) for all and from 97.2 to 99.3% (RR = 1.02, 95% CI 0.99, 1.05) for those who had lived in a sprayed home. When asked about the distance they should stand from the home while spraying, the proportion answering correctly (a few metres away) went from 49.4% pre-performance to 62.0% post-performance (RR = 1.26, 95% CI 1.13, 1.41) for all and 48.9% to 59.1% (RR = 1.24, 95% CI 1.06, 1.44) for occupants of homes ever sprayed. When asked how soon they should wait to re-enter their homes after spraying, 58.5% gave the correct answer (at least an hour) before the presentation but 91.1% did so afterwards (RR = 1.54, 95% CI 1.35, 1.77) for the entire group and increased from 57.1 to 91.9% (RR = 1.61, 95% CI 1.42, 1.82) for occupants of homes that had ever been sprayed.

**Table 2:** Proportion of correct responses to questions about home spraying: pre- and post- performance, Limpopo South Africa, 2015–2016

Question	Correct response	All (% correct)			With sprayed homes (% correct)		
		Pre	Post	RR (95% CI)^a^	Pre	Post	RR (95% CI)^a^
Better to allow spraying for malaria control in home?	Yes	95.1	98.1	1.03 (1.00, 1.06)*	97.2	99.3	1.02 (0.99, 1.05)
How close do you think you should be to your home while it is being sprayed?	A few meters away	49.4	62.0	1.26 (1.13, 1.41)*	48.9	59.1	1.24 (1.06, 1.44)*
How soon after a spraying do you think it is okay to enter your home?	After at least an hour	58.5	91.1	1.54 (1.35, 1.77)*	57.1	91.9	1.61 (1.42, 1.82)*

*p < 0.05

^a^Adjusted for sex and age, using mixed-effects models with a random intercept for village and attendee

Table 3 presents overall summaries of knowledge before and after the presentation. The rate of correct answers for precautions to take before spraying improved from a mean of 59.9% before the presentation to 72.4% afterwards (β = 12.8%, 95% CI 11.0, 14.7) for all participants and from a mean of 60.6 to 73.8% (β = 13.3%, 95% CI 10.5, 16.1) for those who lived in sprayed homes. Correct responses for post-spraying precautions increased significantly from 56.5% before the presentation to 67.7% afterwards (β = 11.6%, 95% CI 9.8, 13.3) for all participants and from 57.5 to 69.4% (β = 12.0%, 95% CI 8.7, 15.3) for those who lived in sprayed homes. Overall correct answers increased from 57.9 to 69.7% (β = 12.1%, 95% CI 10.9, 13.4) for all participants and from 58.8 to 71.3% (β = 12.5%, 95% CI 9.6, 15.4) for those who lived in sprayed homes.

**Table 3:** Comparison of percent correct to all pre- and post-performance spraying precaution questions and for all precautions

Precaution	Maximum	All (% correct)			With sprayed homes (% correct)		
		Pre	Post	β (95% CI)^a^	Pre	Post	β (95% CI)^a^
Pre-spraying	9	59.9	72.4	12.8 (11.0, 14.7)*	60.6	73.8	13.3 (10.5, 16.1)*
Post-spraying	12	56.5	67.7	11.6 (9.8, 13.3)*	57.5	69.4	12.0 (8.7, 15.3)*
All precautions	21	57.9	69.7	12.1 (10.9, 13.4)*	58.8	71.3	12.5 (9.6, 15.4)*

*p < 0.05

^a^Models adjusted for sex and age, using mixed-effects models with a random intercept for village and attendee

The knowledge of attendees for individual pre-spraying precautions significantly improved post-presentation for many, but not for all, of the key items recommended by the WHO. In particular, significantly increased knowledge was observed for removing food from the home, closing cupboards, removing bedding, covering immoveable items with a plastic sheet, and leading animals away from the home (see Table 4). There was no significant improvement in knowledge to remove drinking water and children’s toys from the home. In addition, there was no change in knowledge for the neutral items that we included (e.g., cleaning and waxing floors). Although there was significant improvement in knowledge about what precautions to take before spraying, even after the presentation less than 70% of the participants knew key precautions to take before IRS, such as closing cupboards, removing bedding, covering immoveable items with plastic, and leading animals away. When considering only respondents living in homes that had previously been sprayed, the results were similar.

**Table 4:** Comparison of responses to pre- and post-spraying precautions: attendees’ likelihood of answering correctly before and after the performance

	Precaution		All (% correct)			Sprayed (% correct)		
			Pre	Post	RR (95% CI)^a^	Pre	Post	RR (95% CI)^a^
Before spraying	Set out tea for the sprayers	N/A^b^	7.1	5.6	0.69 (0.40, 1.18)	5.4	2.3	0.40 (0.19, 0.83)^*^
	Remove all food from the home	+	74.0	82.6	1.11 (1.05, 1.17)*	76.6	84.9	1.11 (1.02, 1.20)*
	Remove drinking water from home	+	68.9	71.6	1.04 (0.96, 1.14)	70.9	74.6	1.05 (0.98, 1.14)
	Leave cupboards open	–	79.4	87.0	1.10 (1.05, 1.15)*	82.6	89.0	1.08 (1.02, 1.14)*
	Close all cupboards	+	33.1	41.2	1.28 (1.07, 1.52)*	33.1	44.8	1.40 (1.19, 1.65)*
	Remove bedding from the home	+	21.5	64.7	3.07 (2.44, 3.86)*	20.7	63.5	3.03 (2.30, 4.00)*
	Clean and wax the floor	N/A	16.7	19.8	1.14 (0.89, 1.47)	17.4	15.4	0.90 (0.66, 1.22)
	Cover items that cannot be moved with fabric	N/A	52.5	51.2	0.97 (0.85, 1.10)	59.5	57.9	0.97 (0.84, 1.12)
	Cover items that cannot be moved with plastic sheet	+	45.4	67.2	1.47 (1.33, 1.62)*	43.1	66.6	1.54 (1.36, 1.74)*
	Lead animals away from the home	+	31.3	54.2	1.77 (1.59, 1.96)*	31.8	56.5	1.74 (1.49, 2.05)*
	Close animals inside the home	−	94.6	91.7	0.97 (0.93, 1.00)	95.7	91.3	0.95 (0.91, 1.00)
	Keep your children’s toys inside	−	90.5	91.4	1.01 (0.98, 1.04)	91.3	92.6	1.02 (0.98, 1.05)
After spraying	Sweep the floor	+	39.7	54.1	1.39 (1.20, 1.62)*	39.1	55.5	1.44 (1.15, 1.79)*
	Feed dead insects to animals	−	93.6	96.1	1.02 (1.01, 1.04)*	96.0	97.7	1.01 (0.99, 1.04)
	Discard dead insects in waste heap	−	59.5	76.5	1.32 (1.25, 1.40)*	60.9	79.6	1.34 (1.24, 1.45)*
	Discard dead insects in latrine	+	44.8	71.8	1.60 (1.47, 1.73)*	45.8	73.2	1.59 (1.41, 1.80)*
	Wash your children’s toys	+	48.3	47.5	0.98 (0.85, 1.14)	54.5	46.8	0.87 (0.78, 0.97)*
	Burn dead insects	+	29.6	46.5	1.57 (1.25, 1.96)*	27.8	52.2	1.82 (1.39, 2.37)*
	Wash furniture and seating left inside with soap and water	+	50.2	54.7	1.10 (0.99, 1.22)	51.5	52.8	1.02 (0.90, 1.16)
	Wash the floor with soap and water	+	52.7	52.4	0.99 (0.93, 1.06)	53.2	51.2	0.97 (0.87, 1.09)
	Wash walls with soap and water	−	77.5	81.6	1.05 (1.01, 1.10)*	78.6	84.3	1.07 (1.00, 1.13)
	Rinse wash rag well before using it again	−	61.1	76.4	1.26 (1.14, 1.38)*	59.2	77.9	1.33 (1.14, 1.55)*
	Discard wash rag after using it	+	32.1	64.7	2.10 (1.75, 2.51)*	30.4	69.2	2.23 (1.68, 2.97)*
	Re-plaster or re-paint or wash walls as soon as possible	−	89.2	89.7	1.00 (0.97, 1.04)	93.0	92.3	0.99 (0.95, 1.04)

*p < 0.05

^a^Models adjusted for sex and age, using mixed-effects models with a random intercept for village and attendee

^b^+ indicates that the behaviour should be performed, − indicates the behaviour should not be performed, and N/A indicates that it would not affect insecticide exposure

Of post-spraying behaviours, knowledge improved for sweeping the floors and proper disposal of dead insects (burning or discarding them in the latrine; not feeding them to animals or discarding them in the waste heap). They showed improvement in knowing to discard washrags after using them rather than rinsing them and using again, as well as not washing the walls with soap and water. However, no improvement was noted in the participants’ knowledge that they should clean children’s toys and wash inside furniture or floors with soap and water. Although there was significant improvement in knowledge about some precautions to take after spraying, less than 70% of the participants knew after the presentation to burn dead insects, sweep and wash their floors, wash indoor furniture, discard washing rags, and to wash children’s toys. When considering only respondents living in homes that they had reported as previously sprayed, the results were similar.

## Discussion

Acceptance of IRS was very high both before and after the skit. However, a brief dramatic presentation and song performed by research study staff, who were not formally trained in the performing arts and wearing simple costumes, was an inexpensive method to increase overall knowledge about ways to prevent undue insecticide exposure from IRS in communities such as those in Limpopo, South Africa. Specifically, knowledge increased after the skit and song about appropriate length of reentry interval and distance of residents from the spraying as well as for a number of precautions recommended by WHO: closing cupboards, removing bedding, covering immoveable items with plastic tarps, and leading animals away from the home before spraying, and proper disposal of dead insects and insecticide-contaminated wash rags after spraying. However, there was no demonstrable increase in knowledge for other measures recommended by WHO, such as removing drinking water from the home before spraying or cleaning children’s toys, inside furniture, or floors after spraying. It is possible that there was no increase in knowledge about washing floors, or that participants may have known that floors should be washed but their homes’ mud or daub floors were not washable.

Use of entertaining skits, song, and other folk media [29] to convey public health messages has been employed as a community engagement method in promoting public health and in preventing disease such as of HIV/AIDS [36–39] or tuberculosis [40]. These methods are often used to reach young, low-literacy, or other traditionally hard-to-reach populations [39, 41]. The Program for Appropriate Technology in Health (PATH) developed an approach in Zambia, using drama for malaria education tailored to local language through skits, songs, and dance [42]. However, only a few previous investigations have evaluated the dramatic arts in malaria education. Specifically, a drama performed by professional actors was employed in 20 Cambodian villages to promote the use of insecticide-treated bed nets (ITN), repellents, and early diagnosis; the villagers reported that drama was their preferred choice of community engagement [29]. Using folk theater (Kalajatha) in rural India, 30 local performance artists performed songs, a drama, and a musical drama focused on the transmission, signs, treatment, and prevention of malaria. After 2 months of performances, households from 5 intervention and 5 control villages were interviewed [28]. Although the intervention respondents significantly gained knowledge compared to controls, they did not show immediate behavioral change. In a study from Ghana [27], teachers trained an intervention group of school-age children with picture charts, posters, dramatization, and song on malaria symptoms, treatment and prevention, who then educated adults in their villages with song, poetry recitals, and drama. Adults in the intervention communities significantly increased their knowledge as well as use of ITN, and children from these communities had lowered parasite prevalence compared to prior to the intervention and to control children. The WHO 2015 operational manual for IRS acknowledged the need for community participation and coordination for effective IRS education, and Atkinson et al. [43], in a review of the literature, concluded that community engagement and participation is an essential component of malaria education and ultimate elimination. Most malaria educational intervention studies have employed intensive and expensive means to inform populations, often with multiple in-person sessions using highly-trained personnel or using multiple media such as radio, posters, and booklets [44]. Overall, most educational interventions have resulted in modest, although in some cases statistically significant, increases in knowledge of malaria prevention, symptoms, or treatment [45]. No previous study has compared the efficacy of the various modalities in engaging communities and in malaria education.

The skit-based educational programme was limited. Although knowledge of participants was compared pre- and post-performance, we did not compare change in the intervention-sprayed communities with a change after a sham presentation in a control group from sprayed communities. Given that the presentation sought to encourage IRS while preventing undue insecticide exposure, not much information about malaria symptomatology, prevention, or treatment was provided. Future dramatic presentations could be supplemented to include more information. In addition, estimates of learning may be biased by non-random selection of who turned in the questionnaire. For example, it is plausible respondents low in literacy did not return the survey, although this group may have most benefited from this form of education. It is also plausible that those who learned the least or left early may not have turned in their questionnaire forms. The evaluation of the success of this programme was also limited in that the increase in knowledge was measured immediately after the presentation. Hence, it is not clear whether the attendees retained the new lessons and, most importantly, whether there was a behavioral change in precautionary practices. Time constraints did not allow for the collection of information on attendees’ confidence in their ability to perform safety measures, or any barriers that may exist to their implementation. Ideally, qualitative data collection should be considered to better understand barriers to implementation. The ultimate indicator of success of the programme would be to demonstrate a decrease in insecticide body burden [45–47].

However, there are a number of strengths of this study, including the large sample size and the low cost of a single brief training using lay presenters, rather than professional actors or singers. The presentations were brought to the communities, so large numbers of people could be educated at one time. Thus, this education programme can be readily reproduced even at a larger country-wide scale relatively cheaply using community members to perform the skit and teach the song. Other media or venues should also be explored such as on radio or in churches. Furthermore, although the entertainment value of the presentation was not formally assessed, anecdotal observation by staff and on recorded videotapes of the audience indicated that the attendees paid attention and many were laughing, clapping throughout, and joining in the song. In these low-income communities, where there is low employment and few entertainment options, the presentation provides an opportunity to provide entertaining health education tailored to the culture.

## Conclusions

A single brief dramatic presentation and song was able to increase knowledge of precautions Limpopo villagers could take to reduce insecticide exposure from IRS and its potential adverse health outcomes. Future studies should evaluate whether this method of education results in sustained knowledge and in reduction in insecticide exposure. This approach to community education is promising and deserves additional study and potential expansion to include general information about malaria prevention, symptomatology, and treatment.

## Data Availability

The datasets used for the analysis can be provided by the corresponding author on reasonable request. Data from individual questionnaires is not available due to privacy reasons.

## Appendix 2: Script of the skit performed by VHEMBE staff

A sample clip from the performance can be found at https://www.youtube.com/watch?v=lvZ1W5ZJM8Y

## Appendix 3: The song sung by VHEMBE staff in TshiVenda after the presentation of the skit

A sample clip from the song performance can be found at https://www.youtube.com/watch?v=S_gbrBxDGys

## Abbreviations

CI: confidence interval
DDE: dichlorodiphenyldichloroethylene
DDT: dichlorodiphenyltrichloroethane
IRS: indoor residual spraying
ITN: insecticide-treated bed nets
MAP: Malaria Awareness Programme
PATH: Program for Appropriate Technology in Health
RR: relative risk
VHEMBE: Venda Health Examination of Mothers, Babies and their Environment
WHO: World Health Organization
